# What Do Spatial Distortions in Patients’ Drawing After Right Brain Damage Teach Us About Space Representation in Art?

**DOI:** 10.3389/fpsyg.2018.01058

**Published:** 2018-06-26

**Authors:** Gilles Rode, Giuseppe Vallar, Eric Chabanat, Patrice Revol, Yves Rossetti

**Affiliations:** ^1^INSERM U1028, CNRS UMR5292, Centre de Recherche en Neurosciences de Lyon (CRNL), Equipe ImpAct, Bron, France; ^2^Service de Médecine Physique et Réadaptation, Hôpital Henry Gabrielle, Hospices Civils de Lyon, Saint-Genis-Laval, France; ^3^Dipartimento di Psicologia, Università degli Studi di Milano-Bicocca, Milan, Italy; ^4^Neuropsychological Laboratory, IRCCS Istituto Auxologico Italiano, Milan, Italy

**Keywords:** space representation, neglect syndrome, hyperschematia, right hemisphere damage, egocentric representation

## Abstract

The right cerebral hemisphere plays a crucial rule in spatial cognition, spanning from perception of elementary features, such as location, color, line orientation or shape to representation of different spaces (3D space, allocentric, egocentric, face, personal, peri-personal, or imaginal). One important aspect of its contribution concerns the perception of space symmetry and the representation of objects and scenes, with reference to the midline or body axis. This representation results from a balance between spatial attention processes depending from the two hemispheres. Healthy participants tend to show a discrete deviation of the midline plane representation toward the left side, that is likely to result from the predominance of the activity of the right cerebral hemisphere, mainly oriented toward the contralateral side of space. The visuospatial abilities of the right hemisphere, especially for the representation of the midline plane are crucially engaged in painting and drawing processes in artists. Interestingly, the distortions created by painters of the Cubism period, characterized by an asymmetry of objects and body representations, a specific enlargement or reduction of parts of space, or even by complex distortions of 3D space are analogous to those classically reported in right-brain-damaged patients (unilateral spatial neglect, hyperschematia, constructional apraxia). Understanding the pathological mechanisms of these representation disorders provides meaningful information to apprehend visual artist creations and esthetic perception of space.

## Introduction

**FIGURE 1:**
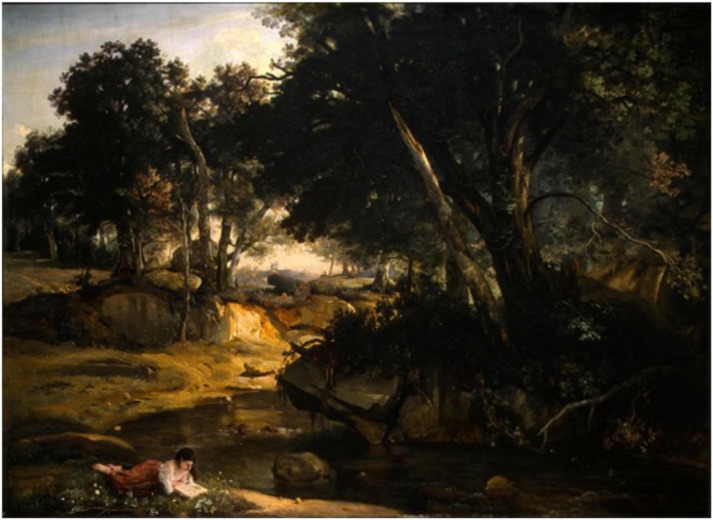
Two types of explicit (verbal and visuospatial) space representation: example of the Fontainebleau forest. Oil painting *La Forêt de Fontainebleau*, par Camille Corot (1833) – Reproduced from National Gallery of Art, Washington DC with permission. Verbal description (in French and in English) by Gustave Flaubert (1821/1880) in the novel “*la vie sentimentale”* 3ème partie chapitre III, page 483. “La diversité des arbres faisait un spectacle changeant. Les hêtres, à l’écorce blanche et lisse, entremêlaient leurs couronnes; des frênes courbaient mollement leurs glauques ramures; dans les cépées de charmes, des houx pareils à du bronze se hérissaient; puis venaient une file de minces bouleaux, inclinés dans des attitudes élégiaques; et les pins, symétriques comme des tuyaux d’orgue, en se balançant continuellement, semblaient chanter”. “The variety of trees furnished a spectacle of the most diversified character. The beeches with their smooth white bark twisted their tops together. Ash trees softly curved their bluish branches. In the tufts of the hornbeams rose up holly stiff as bronze. Then came a row of thin birches, bent into elegiac attitudes; and the pine-trees, symmetrical as organ pipes, seemed to be singing a song as they swayed to and fro”.

One popular distinction for the internal representations of the surrounding world in humans is between verbal and visuo-spatial dimensions (see, e.g., Struiksma et al., 2009). The former mainly depends on the left cerebral hemisphere which is involved in linguistic processes. The latter is mainly related to the activity of the right cerebral hemisphere, which plays a crucial rule in spatial cognition, spanning from perception of elementary features, such as location, color, line orientation or shape to representation of different spaces (Devinsky, 2009; Koch et al., 2013; Zaidel, 2013; Gainotti, 2014; for reviews of different aspects of the processes related to the activity of the right hemisphere, and to the disorders brought about by right hemispheric damage). However, these two types of representation are closely linked: for example, when reading the verbal description of a natural space, as for example the description of the Fontainebleau forest by Gustave Flaubert (1821/1880) in his famous novel entitled ‘*la vie sentimentale’*, we can spontaneously create an inner visual image of the forest which contributes to reinforce the poetic dimension of the narration. In the same way, when looking at an oil-painting of the Fontainebleau forest made by a French painter of the Barbizon school, Camille Corot (1796/1875), in the same era, we could name and identify the different space parts of the image (see **Figure 1**). This distinction between two types of explicit space representation for perception – verbal and visuospatial – is also relevant for the clinical description of cognitive deficits due to focal damage of the brain, especially following a damage of the right hemisphere, where different deficits could be described. These deficits are evidenced in tasks of drawing from memory or by copy. They may be omissions, additions or distortions of parts of the object or the entire object.

## Spatial Distortion After Right Brain Lesion

One interesting feature of misrepresentation concerns the perception of size (and volume) and the midline axis (Milner and Harvey, 1995; Milner et al., 1998). These distortions can be characterized by a size reduction, up to a total omission, of the left part of an object drawn, as illustrated by the object-centered (or allocentric) deficit of left spatial neglect. Sometimes, an expansion of the right hand-side of the object drawn, when productive manifestations with graphic perseverations and/or hypergraphia are also present (Rusconi et al., 2002; Ronchi et al., 2009). This size (and volume) asymmetry may reflect the combination of two underlying disorders of the unilateral neglect syndrome: an ipsilesional bias of the orientation of spatial attention, and an impaired building and/or exploration of internal representations of space (Bisiach et al., 1994; Vallar, 1998; Ricci et al., 2000, 2004; Rode et al., 2017).

**FIGURE 2:**
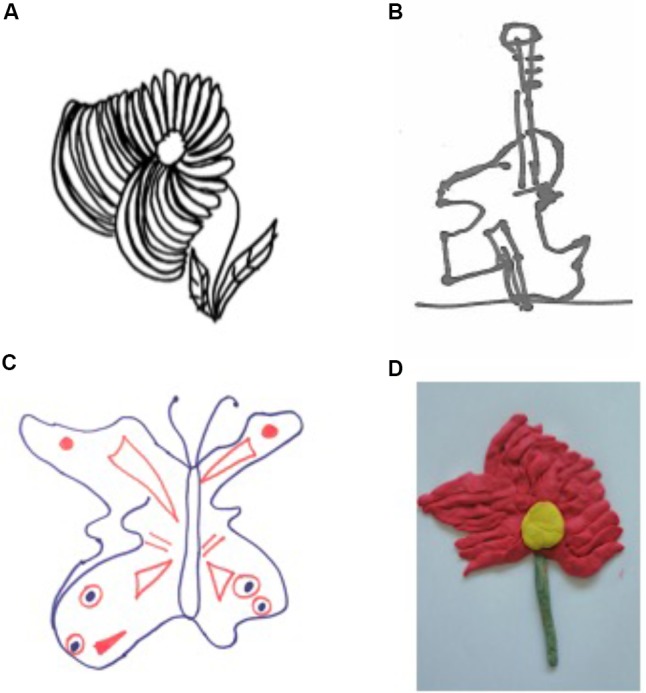
Asymmetry of size (and volume) of objects in drawings of a daisy **(A)**, a guitar **(B)** and a butterfly **(C)** and in modeling a daisy from memory **(D)** in patients with left Hyperschematia (Rode et al., 2014). Patients showed a contralesional productive behavior, characterized by systematic left-sided expansion of drawings and addition of more left-sided details to the drawn object, contralateral to the side of the lesion (daisy).

**FIGURE 3:**
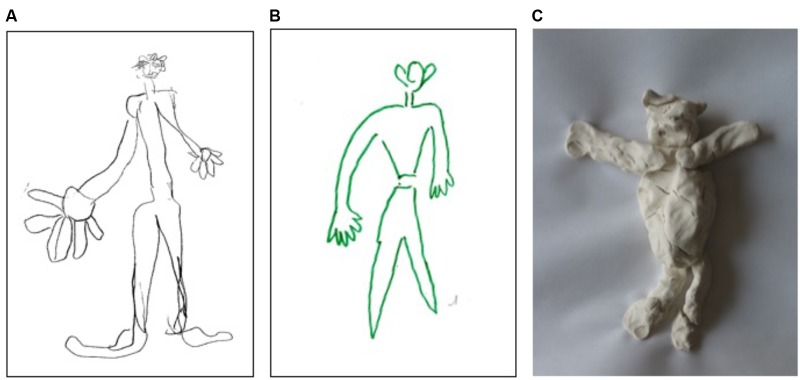
Asymmetry of size (and volume) of objects in drawings of a man from memory **(A,B)** and in modeling a man from memory **(C)** in patients with left hyperschematia. Patients showed a left-hand side enlargement concerning all drawn or modeled body parts: head, arm, chest, and leg.

In others cases, this asymmetry of size (and volume) can be characterized by a systematic enlargement of the left-hand side of objects drawn from memory and by copy. This much infrequent deficit is observed in right-brain-damaged patients, regardless of the presence of the unilateral neglect syndrome, and has been termed “hyperschematia” (Rode et al., 2006). In a previous study, we have reported a series of 7 right-brain-damaged patients showing this spatial distortion for left extra-personal space. Hyperschematia can affect drawing of many different objects as such as a daisy, a tree, a butterfly, a man, a house or scene, the whole of an object, but also some parts of it (Rode et al., 2014). Hyperschematia can also evidenced in three-dimensional (modeling), visuo-contructional tasks, reinforcing the mainly spatial nature of the deficit (Rode et al., 2008) (see **Figures 2, 3**). The disorder remained unchanged when patients were asked to draw in a blindfold condition (Rode et al., 2006). Hyperschematia is most frequently contralateral to a lesion of the right hemisphere, and as for left spatial neglect, (Ronchi et al., 2014). Patients are not aware of the disorder.

**FIGURE 4:**
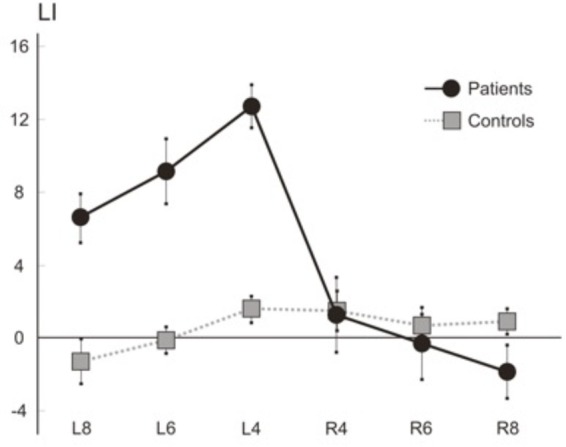
Line extension task. Mean (SEM) LIs of the seven right brain patients and six control participants for rightward (R) and leftward (L) extended lines (length: 4, 6, and 8 cm). The subject had to reproduce the length of a horizontal line in two rightward and leftward movement conditions. In each condition, the end of the line was aligned with the mid-sagittal plane of the body, and the patient reproduced the perceived length of the segment with a rightward or leftward extension. The stimuli were horizontal twenty-four black lines, 1 mm in width, with eight lines for three lengths (4, 6, and 8 cm). The length of the segment drawn by each subject on each trial was measured to the nearest mm. For the leftward extension of each line drawing, a following laterality index score (LI) was computed: Leftward extended length minus length of the right-sided line/leftward extended length plus length of the right-sided line × 100. For the rightward extension of each line drawing, the LI was: Rightward extended length minus length of the left-sided line/rightward extended length plus length of the left-sided line × 100. Positive/negative values of the LI indicate over/under-extension. Mean LIs were computed for the two directions of extension (rightward, leftward), and for three line lengths (4, 6, and 8 cm). All seven patients and six controls were tested. The Figure showed that 7 patients make a leftward overextension for three line lengths (4, 6, and 8 cm) while laterality scores for rightward extension do not differ from those of controls.

Importantly, when patients were asked to perform an estimation of the two horizontal length of two rectangles, by perceptual matching, the comparison of the performances of seven patients with left hyperschematia and controls revealed only a trend toward significance. However, the analysis of the individual scores of patients indicates that only four out of the seven patients showed a rightward overestimation; two patients performed as the control group of neurologically unimpaired participants, and one patient presented with an opposite pattern, namely a leftward overestimation. A disorder of perceptual estimation of lateral extent cannot account for hyperschematia in drawing, being possibly instead an associated deficit with no direct causal relationships with hyperschematia. Conversely, when patients were asked to reproduce the length of a horizontal segment (line extension task), the comparison of the performances of seven patients and controls showed a significant overextension in leftward line extension, while rightward line extension was within the normal range (see **Figure 4**).

**FIGURE 5:**
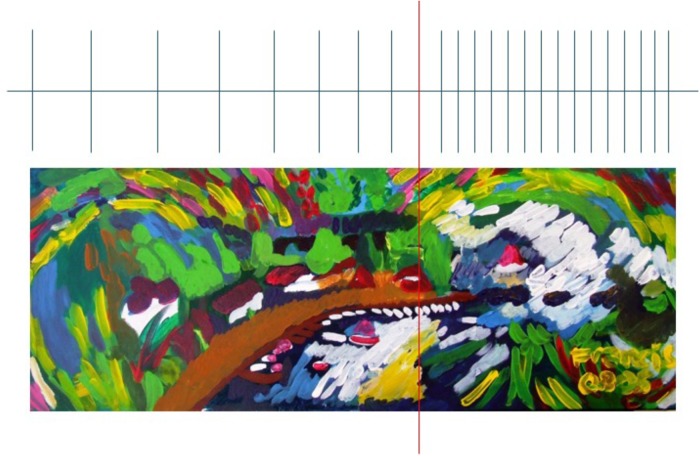
Oil painting from memory of a right brain damaged patient.

These findings suggested that hyperschematia occurs when operations (including planning and execution of motor acts in the left side of space) are required. Moreover they suggest that this non modality-specific distortion of space representation could be due to a lateral anisometry of the internal representation of space, namely: the spatial medium with a leftward relaxation (Rode et al., 2006, 2008). This leftward relaxation of spatial representation can be illustrated by this oil painting made by a right-brain-damaged patient (see **Figure 5** and also Rode et al., 2014).

More generally, a disproportionate enlargement of space representation may also affect bodily space. The first description was provided by the French otolaryngologist Pierre Bonnier (1905), based on the clinical observation of patients with vestibular disorders of peripheral origin (for an historical note see Vallar and Rode, 2009: see also Bonnier’s, 1905 book “Le vertige”). Bonnier reported that these patients may subjectively feel that their body or body-parts are disproportionately enlarged, and interpreted this disorder as a pathologic expansion of the spatial representation of the body or body parts (hyperschematia) (Vallar and Papagno, 2003). These feelings also resemble those reported by patients with macrosomatognosia or asomatognosia hallucinations, with patients referring the feeling that one or more parts of their body are disproportionately larger (Lhermitte and Tchehrazi, 1937; Frederiks, 1969; Denes, 1989), after paroxysmal cerebral disorders such as epilepsy, migraine and hypnagogic hallucinations (Kew et al., 1998; Podoll and Robinson, 2002), somatosensory loss consecutive to a local anesthesia (Gandevia and Phegan, 1999; Paqueron et al., 2003), or a brainstem lesion (Rode et al., 2012). But, unlike hyperschematia for extra-personal space, patients are always aware of their deficit, suggesting different underlying neural and functional mechanisms and neural processes for disorders affecting the representation of the size (and the volume) of a portion of extra-personal or personal spaces.

## Spatial Distortion in Painting

The visuospatial abilities of the right hemisphere, especially those dedicated to the representation of size (and volume) and the midline plane, are crucially involved in painting and drawing processes in artists. Many artists have been interested in the perception and representation of space from the Renaissance to modern times. At the beginning of the twentieth century (1907), Georges Braque and Pablo Picasso created a new artistic movement “Cubism,” which proposed a new approach to the perception and representation of space. Cubism was a revolution in painting and sculpture, and also influenced architecture, literature and music. Their goal was to deconstruct the Euclidean space and to materialize new alternative representations. This new space of cubists was characterized by a substitution of the frozen perspective of classical painters by a moving perspective. The discovery of cubists has been to show all objects from all sides at once, i.e. merging several viewpoints (Paulhan, 1993).

**FIGURE 6:**
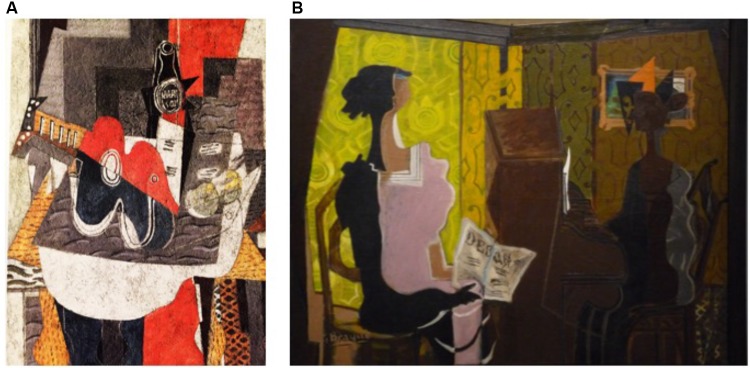
Oil paintings of Georges Braque. “*Guitare et bouteille de marc sur une table”* (1930) – Reproduced from Cleveland Museum of Art with permission **(A)**; “Le duo” (1937) – Reproduced from photo aaa Centre Pompidou, MNAM-CCI, Dist. RMN-Grand Palais/Philippe Migeat with permission **(B)**.

This deconstruction of space concerned the symmetry of size (and volume) and the representation of the plane midline. It can be illustrated by the oil painting intitled “Guitare et bouteille de marc sur une table” (Braque, 1930), which is a still life composed of several objects placed on a pedestal table. In addition to removing all coherent perspectives, the shape of the represented objects is asymmetrical; for example, for the guitar, the left half of the widened box is enlarged contrasting with an enlargement of the opposite part of the rose and the neck (see **Figure 6A**). These asymmetries of size and volume contribute to creating distortions in the representation of space and object, and thus materialize a new space in Braque’s own words: ‘*What attracted me greatly and which was the main direction of Cubism was the materialization of this new space that I felt. So I began to do mostly still-life, because in nature, there is a tactile space, I will say almost manual. It was this space that attracted me a lot. For this was the first Cubist painting, the search for space. “*G. Braque. (cited in: Vallier, 1954).

G. Braque also applied this new materialization of space to the bodily space. The oil painting untitled “Le Duo” painted in 1937, is an illustration. Braque represented two persons in front of an upright piano. The body of the woman sitting in front of the piano is unstructured; the bodily space is separated into two asymmetrical halves: an enlarged left hemi-body seen from the front and in the light and a shrunken right hemi-body seen in profile and in the shadow (see **Figure 6B**). This deconstruction-reconstruction of the bodily space and the distortions that are attached to it allowed Georges Braque to represent all the possible perspectives of the bodily space on the same plane with a displacement of the middle axis of the body. These contribute to create a new esthetic representation of bodily space that Braque will further deepen in his subsequent works.

It is interesting to compare these distortions with the spatial distortions produced by patients with hyperschematia. In these two a priori remote situations, there is a loss of the symmetry of representation of size and volume and a displacement of the representation of the middle axis. In G. Braque, these distortions are the result of a creative and conscious process, while the “hyper schematic” ones result from an unconscious process as suggested by the patients’ unawareness of the disorder. Nevertheless, in both situations, the result consists in the building up of an asymmetric representation of space and body midline.

However, these two types of distortions ask about the neural processes underlying the building of these representations of size, volume, and bodily space. These result from a balance between spatial attention processes of the two hemispheres. Healthy participants tend to show a discrete deviation of the midline plane representation toward the left side, reflecting the predominance of the right cerebral hemisphere activity for the contralateral space. This predominance is dramatically reduced after damage of the right hemisphere producing various distortions of extrapersonal and bodily space (Irving-Bell et al., 1999; Vallar and Bolognini, 2014). Hyperschematia constitutes an uncommon example of a distortion affecting size (and volume) and space. The disorder is most frequently contralateral to the right hemispheric lesion, as left spatial neglect. However, a few reports of ipsilesional right hyperschematia (Saj et al., 2011), and of ipsilesional right overextension (Bisiach et al., 1998) have been reported. Overall, these distortions suggest that the unbalance between the spatial processes of the two hemispheres is not confined to an ipsilesional orientational bias of spatial attention, that may account for the defective manifestations of spatial neglect but also concerns the representational dimension (Bisiach et al., 1996; Savazzi et al., 2007; Manfredini et al., 2013; Vallar and Bolognini, 2014).

Similar neural processes may be at work during artistic creation, reinforcing the idea that one intriguing aspect of the ‘spatial brain’ is its capability to construct non-Euclidian representations of extrapersonal and bodily spaces. This widening of the left side spatial representation can be seen as an alteration of the internal function of measuring distances, also called the metric in mathematics. The patient would then present a non-constant right-to-left metric that would increase perceived distances in the controlesional side. Unconsciously, the patient distributes his painting homogeneously in a reference system whose calculation of distances undergoes a transformation gradient on a right / left axis. According to the same scheme, Cubist artists would make the voluntary choice, based on a more or less conscious evaluation of space, of modifying the metric of their drawing locally on its elements and thus produce multiple deformations depending on the position on the final work similarly as they do to encode depth in their paintings.

## Disclosure

All authors have full access to all the data in the study and take responsibility for the integrity of the data and the accuracy of the data analysis.

## Author Contributions

GR designed the work acquired, analyzed, did interpretation of data for the work, and drafted the work. GV and YR did interpretation of data for the work and revised the work critically for important intellectual content. EC revised the work critically for important intellectual content. PR analyzed data for the work and revised the work critically for important intellectual content.

## Conflict of Interest Statement

The authors declare that the research was conducted in the absence of any commercial or financial relationships that could be construed as a potential conflict of interest.
